# Microphysical Features of Rain and Rain events during different Seasons over a Tropical Mountain location using an Optical Disdrometer

**DOI:** 10.1038/s41598-019-55583-z

**Published:** 2019-12-13

**Authors:** T. S. Sreekanth, Hamza Varikoden, G. Mohan Kumar, E. A. Resmi

**Affiliations:** 1 National Centre for Earth Science Studies, Thiruvananthapuram, 695011 India; 2 Indian Institute of Tropical Meteorology, Pashan, Pune 411008 India

**Keywords:** Atmospheric dynamics, Hydrology

## Abstract

In the present study, seven-year-long observations of rain microphysical properties are presented using a ground-based disdrometer located at Braemore; a site on the windward slope of the Western Ghats (WG) over the Indian Peninsula. The annual cycle of rainfall shows a bimodal distribution with a primary peak during summer monsoon and secondary peak during pre-monsoon. Pre-monsoon rain events are less in number but are with high intensity and characterize large raindrops and low number concentration. During summer monsoon, short and less intense rain events with small drops are noticed. Post-monsoon rain is having a long duration less intense events with lower concentration of large raindrops compared to the summer monsoon. In the seasonal variation of mean diameter (D_m_) and raindrop concentration (N_T_) with Rain Intensity (RI), winter and pre-monsoon rains exhibit higher values of D_m_ and lower values of N_T_ compared to the summer and post-monsoon seasons for all the RI ranges. The mean features of the rain microphysical parameters are also supported by the case studies of rain events. RI, D_m_ and N_T_ are categorized into different range bins for all the seasons to identify their variation and relative rainfall contribution to the total seasonal rainfall. Heavy drizzle/Light rain has maximum rain duration, and the relative contribution to the rainfall is high from heavy rain type. Winter and pre-monsoon rains are mostly contributed from the larger raindrops (>D_m_3), and during summer and post-monsoons it is from D_m_2 onwards. The distribution of occurrence frequency of N_T_ and rainfall are similar during all four seasons. N_T_2 recorded rainfall percentage nearly the same as N_T_1 in summer monsoon and this also supports large number of raindrops in this season. In RI-Duration analysis, all seasons showed similar distribution, and 90% of total duration is contributed from RI with less than 20 mm h^−1^.

## Introduction

Cloud microphysical processes play vital roles in the genesis and modulation of stratiform/convective rain, especially over mountain regions. Different microphysical properties induce considerable differences in intensity and distribution of rain^1,2^. Increased precipitation due to forced updraft in the windward side and suppressed rainfall in the leeward side is caused by the mountain ranges^3^. Climatic mountain features differ substantially over short distance as they have complex topographies^4,5^. In the Indian Peninsula, the west coastal region receives maximum rainfall^6^ due to the orographic lifting over the Western Ghats (WG) mountain ranges^7^. The WG runs parallel to the west coast of the Indian Peninsula from 8.5° to 21° N by maintaining a mean height of 800 m^8^ with multiple peaks exceeding 2500 m. Even though the WG is not very high in altitude, it plays a vital role in convective process during the Indian summer monsoon^9^. Topographic variations play an important part in triggering cloud bands due to orographic lifting of the low level westerlies, that causes rainfall and micro-scale topographic features^10–15^. Earlier studies reported that the WG also influences the regional orographic precipitation along the west coast of India on the basis of *in-situ*, model and satellite observations^3,6,7,16,17^.

Observational evidences indicate that shallow organised and extended orographic convective cloud structures may occur at intermediate heights of mountain ranges^18^. These convective organisations have substantial impacts on regional rainfall and thus can strongly influence the long-term trends, especially in the mountainous regions. Studies^19,20^ conducted in the Southern Appalachian mountains also showed high variability in rain microphysics associated with orography. Experiments at Dominica (15°N, 61^o^W)^21^ concluded that the convection there is caused by terrain forced ascent and this terrain also influenced the dissipating phase of rain events. In another experiment at the same site, they identified the role of trade winds in forming thermal and mechanical convections^22^. In experiments over tropical islands, it is found that rain frequency and total rainfall are significantly high over large islands compared to the surrounding oceans^23^. They attributed the changes in rainfall to the strength of orography associated with the large islands. A similar study over Peninsular Malaysia^24,25^ also found that the lack of diurnal cycle of orographic rain is attributed to enforced upslope flow rather than elevated surface heating. From raindrop size distribution (DSD) studies at tropics^26^ distinct differences are observed between orographic and non-orographic rain events and they found that more number of massive drops are dominated in high Rain Intensity (RI) in the orographic case. A comparative study of rain between the stations in the east and west coasts of India unravelled the differences in the DSD^27^.

A field study that focused on the impacts of rainfall properties on soil erosion over steep slopes found that a decreased rate of infiltration due to the sealing of soil pores by large raindrops^28^. In a laboratory experiment with different RI (60, 90, 120 mm h^−1^) on a 15^o^ slope, the roughness of the entire slope was found to be altered with RI^29^. In soil splash erosion studies, size and velocity information of raindrops are required to estimate kinetic energy as a driving force of soil detachment and mobilization^30^. The fall speed of raindrops is directly proportional to the height of the topography and this topography also influences rainfall kinetic energy in order to impact the soil^31,32^. All these demand more work on details of rainfall events and the microphysics of rain such as raindrop size, fall speed and RI distribution at high altitude sites to identify the soil loss during rain to reveal the underlying processes.

**Figure 1:**
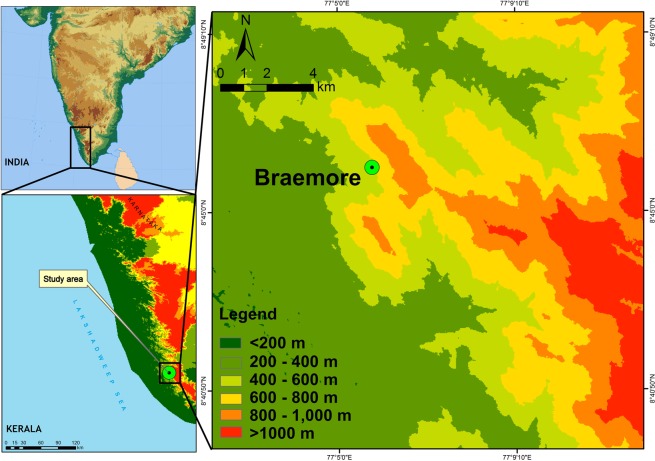
Location map with elevation – Braemore station. (Plotted in ArcMap).

The location map of the measurement site: Braemore (8°45′N, 77°5′E, 400 m AMSL), is given in Fig. 1, the site is located at the southern Peninsular India (the Kerala and Indian Peninsula are given in the insets of the figure). This measurement site is situated on windward side of the WG at a radial distance of about 40 km from the Arabian Sea coast line. The slope of the WG increases gradually in the direction of the sea breeze from 5° to the horizontal at 20 km inside the coast, to 30° to the horizontal at 45 km^33^, there the altitude increases to 400 m^14^. The role of mountain weather in convective Cb formation at this site was investigated^34^ and found strong updrafts as a characteristic of the mountain weather during thunderstorm months (April, May, October and November). Atmospheric instability plays a key role in governing mountain weather conditions. Thunderstorm formation associated with detected instabilities and updrafts is already reported from this site^14,34,35^. The influences of terrain slope and arrival of sea breeze are reported for the formation of orographic convective thunderclouds over the mountain slopes of the Ibuki Mountains, Japan and South Brazil^36,37^. Studies on microphysical properties of cloud and rainfall on leeward (Pune) and windward (Mahabaleshwar) sides of the WG also confirm the role of orography in mountain regimes^38^. The study from Mahabaleshwar also reported that the shallow convective rain with small raindrops contributes significantly to the total rainfall^39^. In mountain regions where orographic rain is dominated, studies on rain microphysics with high frequency data are not adequately investigated. Analyses of DSD parameters such as RI, N_T_ and D_m_ from mountain areas are rare, and they are crucial in region-specific rain modelling, soil erosion and hydrological applications. This study is intended for a better understanding of orographic rain microphysics. During the four seasons, statistical parameters like maxima, mean and standard deviations (SD) are computed for the microphysical parameters (RI, D_m_ and N_T_) of rainfall and observed considerable variations in these parameters.

## Data and Methodology

### Data and instrumentation

The DSD was measured using a ground based PARSIVEL (Particle Size Velocity) disdrometer, which was installed and operational at the site, Braemore. The data is collected at 1-minute interval with the assumption that the dynamics of rain might not alter within this interval and can coverup the uncertainties in DSD^39^. The DSD derived microphysical parameters (RI, D_m_, and N_T_) are calculated from this one-minute data. Further, a double layer screening is applied to this data set such that the samples with RI > 0.1 mm h^−1^ and N_T_ > 10 m^−3^ are considered for the present study and the rest of the data are screened-out. From this, rain events with less than 5-minute duration are also screened-out to obtain a more consistent data set. These quality checks are subjected to the dataset after removing the ‘margin fallers’^40–42^ from the original dataset. The screened-out samples from the analysis are considered as trace rains and its percentage of duration is computed for each month. This quality-controlled data from seven years (2012–2018) are categorised into four different seasons, namely: Winter (January & February), Pre-monsoon (March to May), Summer monsoon (June to September) and Post-monsoon (October to December). Seasons are chosen as per the India Meteorological Department’s classification of monsoon seasons (http://www.imd.gov.in). Season-wise data availability is given in Table 1. Rain events are identified and computed their duration and accumulated water (rainfall) for all the seasons.

**Table 1:** Availability of data at Braemore.

Year	Winter	Pre-monsoon	Summer monsoon	Post-monsoon
2012	N	Y	Y	Y
2013	Y	Y	Y	Y
2014	Y	Y	Y	N
2015	Y	Y	Y	Y
2016	Y	Y	N	N
2017	N	Y	Y	Y
2018	Y	N	N	N

Note: N-data not available; Y-data available.

#### PARSIVEL Disdrometer

The PARSIVEL disdrometer^30,40,43,44^ is an optical sensor, which is designed to measure the hydrometeor fall speed up to 20 ms^−1^ and diameter up to 24.5 mm in 32 velocity and size classes, respectively. This ground-based instrument transmits a laser beam of width 30 mm and of length 180 mm at a wavelength of 910 nm from a photo transmitter. A photodiode sampling at 50 kHz receives the laser light and a resultant voltage is produced. This horizontal setup of sampling area of 54 cm^2^ is kept at 1 m height perpendicular to flat concrete surface. When a raindrop crosses the sampling area, a portion of the transmitted light is blocked and a voltage drop is produced. The amplitude and duration of this voltage drop are measures of the size and fall speed of the crossed raindrop, respectively. More details on data quality, size & velocity classes, and technical details can be found in previously published articles^41,45–47^. The sampling area of PARSIVEL can be estimated as a function of raindrop diameter and hence the effective sampling area is reduced when it detects the raindrops at margins due to the exclusion of ‘margin fallers’. The sampling area for the *i*^th^ class^40–42^ is

1 $${S}_{i}^{Pars}={10}^{6}\times L[B-\frac{{D}_{i}}{2}]$$

where ${S}_{i}^{pars}$(m^2^) is the sampling area, D_i_ (mm) is the class-centre equivolume drop diameter for the *i*^th^ diameter class, L and B are length and width of the laser beam, respectively.

### Methodology

**Figure 2:**
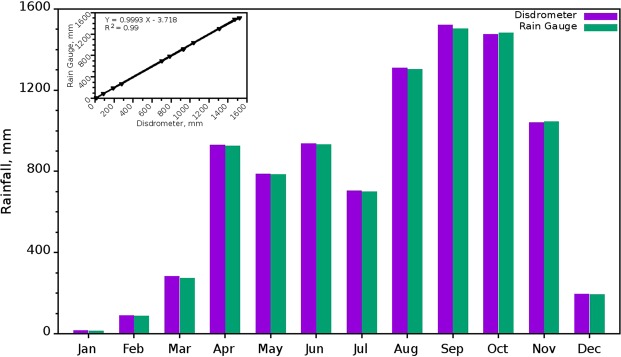
Annual distribution of rainfall for the study period based on disdrometer and manual rain gauge. The inset graph shows the correlation between the two observations. (Plotted in Gnuplot).

Daily rainfall from disdrometer and manual rain gauge from the site is compared and their annual cycle are analysed during the study period (Fig. 2). Rain events are identified from the seven year data and estimated the rainfall amount, rain duration and the number of rain events for the four seasons. Mean of RI, duration per event and rainfall per event is also calculated from rainfall, rain duration and number of rain events, respectively. Statistical analyses of the microphysical parameters (RI, D_m_ & N_T_) and Box-Whisker analysis of rainfall per event and rain duration per event are also carried out.

RI, D_m_ and N_T_ are computed by using Eqs. (2)–(6) ^48–50^. The rain events are identified based on its start and end times. The duration (minutes) and rainfall (mm) are also calculated for each rain event from the PARSIVEL data.

The equation of DSD moment as

2 $${M}_{n}={\int }_{0}^{\infty }N(D){D}^{n}{\rm{dD}}$$

where n stands for moment number, D is the raindrop diameter and N(D) dD is the number of raindrops per unit volume with diameters between D and D + dD

RI and number density of raindrops of the diameter corresponding to size class i per unit volume N(D), is computed using Eqs. (3) and (4), respectively.

3 $$RI=\frac{6\pi }{{10}^{4}}\mathop{\sum }\limits_{i=1}^{32}\mathop{\sum }\limits_{j=1}^{32}{V}_{j}N({D}_{i}){D}_{i}^{3}\Delta {D}_{i}\,$$

4 $$N({D}_{i})=\mathop{\sum }\limits_{j=1}^{32}\frac{{n}_{ij}}{A.t.{V}_{j}.{D}_{i}}$$

where *n*_*ij*_ is the number of drops counted in the size bin i and velocity bin j, A is the sampling area in m^2^, t is the sampling interval in seconds, V_j_ is the fall speed of velocity bin *j* in ms^−1^ and ΔD_i_ is the corresponding diameter interval in mm^51^.

D_m_ and N_T_ are calculated using Eqs. (5) and (6), respectively.

5 $${D}_{m}=\frac{{M}_{4}}{{M}_{3}}$$

6 $${N}_{T}=\mathop{\sum }\limits_{{\rm{i}}=1}^{32}N({D}_{i}){\Delta {\rm{D}}}_{i}$$

For further analysis of rain, the DSD parameters (RI, D_m_ & N_T_) are divided into six different bins. For each bin, the percentage of occurrence, amount of rainfall and percentage of contribution to the total rainfall is computed. For this, RI is classified into six categories of which the first five are as per the World Meteorological Organization (WMO) norms (Guide to Meteorological Instruments and Methods of Observation, 2008). An additional bin is added to include “very violent” rain type (RI > 100 mm h^−1^), which is very frequent over the study region. The D_m_ and N_T_ are also categorized into 6 bins of 1 mm and 1000 drops m^−3^ bin widths, respectively. The different categories of RI, D_m_ and N_T_ are given in Table 2.

**Table 2:** Categories of RI, D_m_ and N_T_.

Rain Intensity, RI			Mean drop diameter, D_m_		Drop concentration, N_T_	
Variable	Range, mm h^−1^	Rain Type	Variable	Range, mm	Variable	Range, m^−3^
R1	0.1–0.5	MD	D_m_1	<1	N_T_1	10–1000
R2	0.5–2.5	HD/LR	D_m_2	1–2	N_T_2	1000–2000
R3	2.5–10	MR	D_m_3	2–3	N_T_3	2000–3000
R4	10–50	HR	D_m_4	3–4	N_T_4	3000–4000
R5	50–100	VR	D_m_5	4–5	N_T_5	4000–5000
R6	>100	VVR	D_m_6	>5	N_T_6	>5000

MD = Moderate Drizzle, HD/LR = Heavy Drizzle/Light Rain, MR = Moderate Rain,

HR = Heavy Rain, VR = Violent Rain and VVR = Very Violent Rain.

## Results and Discussions

### Rainfall distribution

In Braemore, rainfall shows distinct differences on the seasonal pattern. Figure 2 shows the inter-comparison of monthly rain recorded using disdrometer and manual rain gauge at the study site. An excellent agreement is found with a correlation coefficient of 0.99 between two independent instruments (disdrometer and manual rain gauge) with negligible aberrations. A bimodal distribution with a primary peak during the summer and secondary peak during pre-monsoon is observed in the multiyear mean of annual rainfall. Enhancements in rainfall can be observed from second half of the summer monsoon to first half of the post-monsoon in the seven-year total rainfall. During April and May, a good amount of rainfall is recorded at this site than that in the first two monsoon months (June and July). In general, copious rainfall is available in this mountain slope during the summer monsoon in addition to thunderstorm seasons (pre- and post- monsoons) and the rainfall is minimum during the winter season.

### Statistical studies on duration and rainfall per events

Seasonal variations of rainfall and rain duration at this mountain slope are described in Table 3. The present study analyzed a total of 3551 rain events, which covers a duration of 77956 minutes (1299.3 hours) with an accumulated water of 9264.77 mm. The least rainfall (101.67 mm), rain duration 1244 (minutes) and number of rain events (80) are found during the winter season and the maximum values are found during the summer season (4463.14 mm for rainfall, 38617 minutes for rain duration and 1910 for number of rain events). Winter season records least in mean duration per event (15.55 minutes), mean rainfall per event (1.27 mm) and mean RI (4.56 mm h^−1^). Maximum mean duration and rainfall per events are found during pre-monsoon. Mean RI (9.34 mm h^−1^) also is high during pre-monsoon.

**Table 3:** Total rain duration, the total number of rain events, total rainfall, mean event duration and mean rainfall per event during the four seasons.

Season	Rainfall (mm)	Total rain duration (min)	No. of events	Mean Duration per event (min)	Mean Rainfall per event (mm)	Mean RI (mm h^−1^)
Winter	101.67	1244	80	15.55	1.27	4.56
Pre-monsoon	1995.29	12423	631	19.69	3.16	9.34
Summer monsoon	4463.14	38617	1910	20.21	2.34	6.79
Post-monsoon	2704.67	25672	930	27.70	2.90	6.18

**Figure 3:**
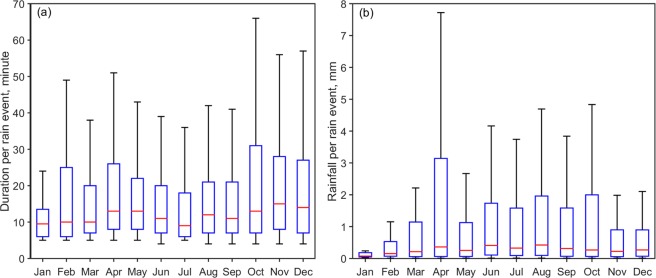
BoxWhisker analysis of (**a**) Duration per rain event and (**b**) Rainfall per rain event during the seven year study period. (Plotted in Matlab).

Box-Whisker analysis (Fig. 3) is done on duration per event and rainfall per event during the study period. This figure depicts the distribution of data based on the five number summary: minimum, first quartile, median, third quartile, and maximum.

During the study period, the median of duration per event increases from January to April and then decreases gradually from May to July. The spread of duration per event is less (high) during the summer (pre- and post-) monsoon seasons indicating more number of short (short and long) duration rain events. From pre-monsoon to summer monsoon, a decrease is observed in third and fourth quartiles of duration per event, whereas it is increased largely from summer monsoon to post-monsoon. Moreover, the spread of events at fourth quartile is more than in the first quartile and the extreme events also show high monthly variation. Similarly, the median of rainfall per event increases gradually from January to April and it shows a peak in April with high variability (Fig. 3b). During summer monsoon and in October, 75% of rainfall lies below 2 mm indicating more consistent and less spread in rainfall amount. During April, 75% of rainfall per event lies below 3 mm and 25% of rainfall lies above 3 mm. Also, from rain duration and rainfall per event, probability of extreme rainfall events is high in April in this mountain slope, which indicates a unique nature of rainfall distribution in Braemore.

**Figure 4:**
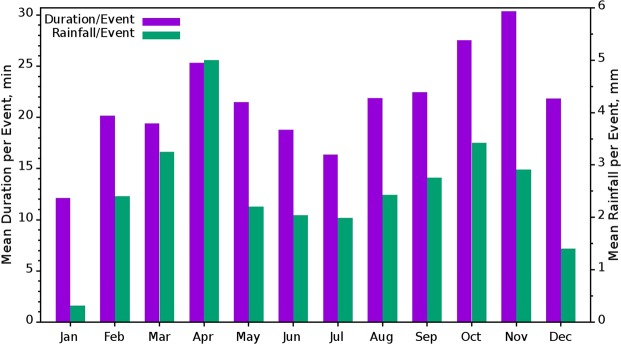
Monthly distribution of mean event duration in minutes and rainfall per event in mm. (Plotted in Gnuplot).

A bimodal variation is observed in the annual distribution of both mean rain duration per event and mean rainfall per event (Fig. 4). For both the parameters, the first peak is in April, however, the second peak is in November and October for mean rain duration per event and mean rainfall per event, respectively. Mean rainfall per event is less in summer monsoon and high in pre-monsoon and followed by post-monsoon. It can be inferred from the rainfall and duration per event that the pre- and post-monsoons rains are intense while comparing with summer monsoon rain. During February, March, April, October and November, rainfall per event is high and this may be due to the development of regional orographic convective clouds and associated rain^37,52^. For a mid-altitude mountainous site, orography plays a pivotal role in increasing water content during convection^26^. This site is already reported as instrumental in the formation of thunderclouds especially during the pre- and post-monsoon seasons^14,34,35^. Due to thunderstorms, rain events are often convective at that time and result in high rainfall content in rain events particularly in April.

**Figure 5:**
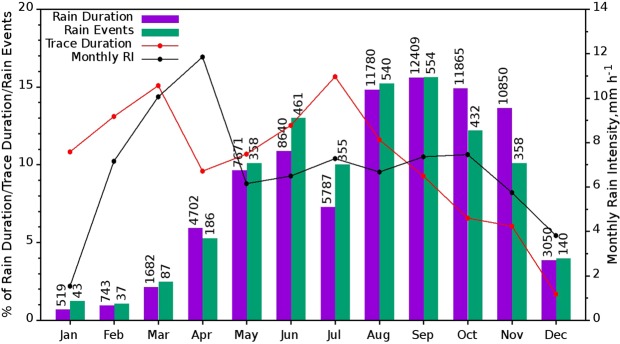
Monthly distribution of percentages of rain, rain events, trace rain duration and mean RI. The numbers over bars are the actual duration in minutes and number of events respectively. (Plotted in Gnuplot).

The monthly distribution of rain parameters is given in Fig. 5. The monthly mean RI is computed from monthly total rainfall and rain duration. Percentage of trace rain duration from each month is calculated and plotted as a solid red line. Percentage of rain duration and rain events shows gradual increment from January to September with a dip in July and a then fast decrease until December. From May to October, mean RI (black line in the figure) is comparatively less with slight fluctuations, and then it decreases till December. Even though rain duration and rain events are less in February to April, monthly mean RI is high. Percentage of trace rains shows an increase from January to March and followed by a decrease in April, with a further increase till July and thereafter a gradual decrease till December. During May, June and July the percentage of rain events exceeds the percentage of monthly rain duration which indicates more number of short period rain events. During the months of thunderstorms (April, October & November), the percentage of rain duration exceeds the percentage of rain events. During the summer monsoon, consistently July registered less rain compared to other months in that season and this also can be confirmed from Fig. 2.

From these analysis on different features of rainfall, it can be concluded that, (a) winter is the least rainfall season, (b) rainfall is intense during February to April while it is less intense during the summer monsoon followed by post-monsoon, (c) more (few) number of short (long) rain events are repeated in summer monsoon (February and pre-monsoon), however, post-monsoon registered more number of long rain events, (d) amount of rainfall from rain events during the summer monsoon and October is more consistent with less variation and (e) percentage of trace rain duration is high in March and July and it is less during the post-monsoon season.

#### Statistical Properties of microphysical parameters RI, D_m_, N_T_ and Daily Rainfall

The statistical properties (maximum, mean and standard deviation) of RI, D_m_, N_T_ and rainfall are described in Table 4. Since rainfall is rare in winter, all statistical parameters during the season has consistently less values and discussions are mainly focused on other seasons. During the study period, RI (daily rainfall) maximum is registered high in post-monsoon followed by pre-monsoon (summer and then pre-monsoon), which indicates strength of rainfall at this site during these seasons. In the case of mean and SD, RI show high values during the pre-monsoon and post-monsoon seasons followed by summer monsoon. In D_m_, pre-monsoon registers high values in mean and SD followed by post and summer monsoons. Mean value of N_T_ records high during the summer monsoon and then followed by pre and post-monsoons. However, the high SD is observed during the pre-monsoon and followed by the summer and post- monsoons. In the case of daily rainfall, the highest value of mean rainfall is observed during post-monsoon and least observed during pre-monsoon. The standard deviation also follows the similar pattern of the mean rainfall.

**Table 4:** Maxima, mean and SD of rain intensity, mean drop diameter, drop number concentration and daily rainfall in Braemore.

Stat. Var.	RI, mm h^−1^				D_m_, mm				N_T_, m^−3^				Daily Rainfall, mm			
	Wint	Pre	Sum	Post	Wint	Pre	Sum	Post	Wint	Pre	Sum	Post	Wint	Pre	Sum	Post
Max	94.70	216.49	194.6	253.05	4.87	5.48	5.47	5.47	2401.5	8998.32	9971.1	9956.8	51.2	125.84	141.1	173.83
Mean	4.56	9.34	6.79	6.18	1.24	1.59	1.27	1.30	280.2	417.87	504.8	395.8	4.48	10.40	15.24	16.38
SD	11.84	20.37	14.34	15.27	0.60	0.74	0.52	0.52	342.7	686.54	580.9	512.1	11.38	17.34	19.94	24.01

High values of mean and SD in RI during pre-monsoon indicate the strength and high variability. The mean and SD of D_m_ reveals the massiveness of raindrop and large variability during the pre-monsoon. Post-monsoon rain also has large raindrops compared to summer monsoon, but with less variability than the pre-monsoon season. During the summer monsoon, consistent small drops are observed and it also evidenced by less D_m_ in both mean and SD. In the case of N_T_, post-monsoon shows less number of raindrops without much variability throughout the season. Pre-monsoon also record less in N_T_ with high variability. During the summer monsoon, high value of mean in N_T_ with comparatively less SD indicates large number of drops throughout the season. The increasing SD in daily rainfall from winter to post-monsoon conveys the increasing variability of daily rainfall throughout the seasons.

From statistical analysis of microphysical parameters and daily rainfall, it can be concluded that (a) intense pre-monsoon rainfall is contributed by massive drops, but with less number concentration (N_T_), with high variability in RI, D_m_ and N_T_, (b) summer monsoon rain is exhibiting large number of small drops and hence less RI than pre-monsoon, with relatively low variability in all the parameters, and (c) post-monsoon rain is also less intense with less number of large raindrops than summer monsoon and with high variability in RI and less variability in D_m_ and N_T_.

### Drop size distribution of different rain types

**Figure 6:**
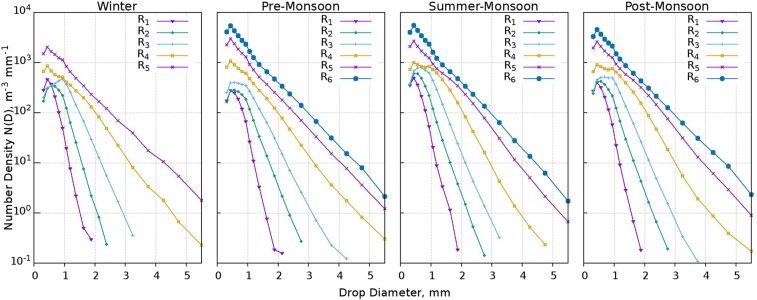
DSD spectrum of different rain types during four seasons Winter, Pre-Monsoon, Summer-Monsoon, Post-Monsoon. There is no rain in the R6 category in winter. (Plotted in Gnuplot).

Figure 6 shows the spectrum of variation in DSD of six different RI bins (R1-R6, Table 2) during all the seasons for the 7-year study period. During the summer monsoon, the raindrop number density (N(D), m^−3^ mm^−1^) in small drop sizes are high and in large drop sizes are less in rain types from R1 to R4 than in the other three seasons. During the pre- and post-monsoons, N(D) in small drops from rain types R1 to R4 are less and more in the case of large drops. In R5 rain type, the winter registered less (more) number of small (large) drops than other three seasons but no rain has occurred in R6 category. With the log scale in ordinate, the increments in low ranges and decrements in high ranges of N(D) are found marginal but they are significant in linear scale. Thus, it can be inferred that pre-monsoon and post-monsoon have more large drops and few small drops when compared with the summer monsoon. The same can be observed in R5 rain type during the winter.

### Variations of N_T_ and D_m_ with RI

**Figure 7:**
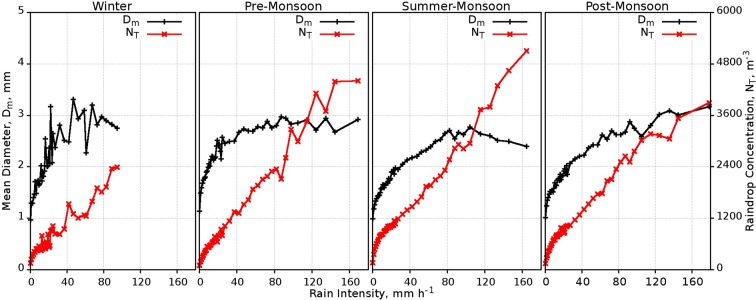
Seasonal variation of D_m_ and N_T_ with respect to RI. (Plotted in Gnuplot).

The variation of D_m_ and N_T_ with RI during the four seasons is depicted in Fig. 7. For up to RI < 80 mm h^−1^, D_m_ shows high values with low values in N_T_ during the winter and pre-monsoon. Beyond 80 mm h^−1^, D_m_ is decreasing (increasing) during the summer (post) monsoon period; however, it remains same during the pre-monsoon period with slight deviations. In contrast to D_m_, N_T_ is increasing during the summer monsoon while its rate of increment is substantially reduced during the post-monsoon. On integrating all the observations, it can be inferred that during winter and pre-monsoon large drops are formed within 80 mm h^−1^, whereas in the post-monsoon season, the large drops are found above 80 mm h^−1^. Massive raindrops are found in the RI of 80–100 mm h^−1^ and decreases thereafter during the summer monsoon, which suggests the number of small-sized drops is more at high RI. All these suggest orographic convection during pre and post monsoon rains at this mountain slope^14,35^.

### Case studies of rain events

**Figure 8:**
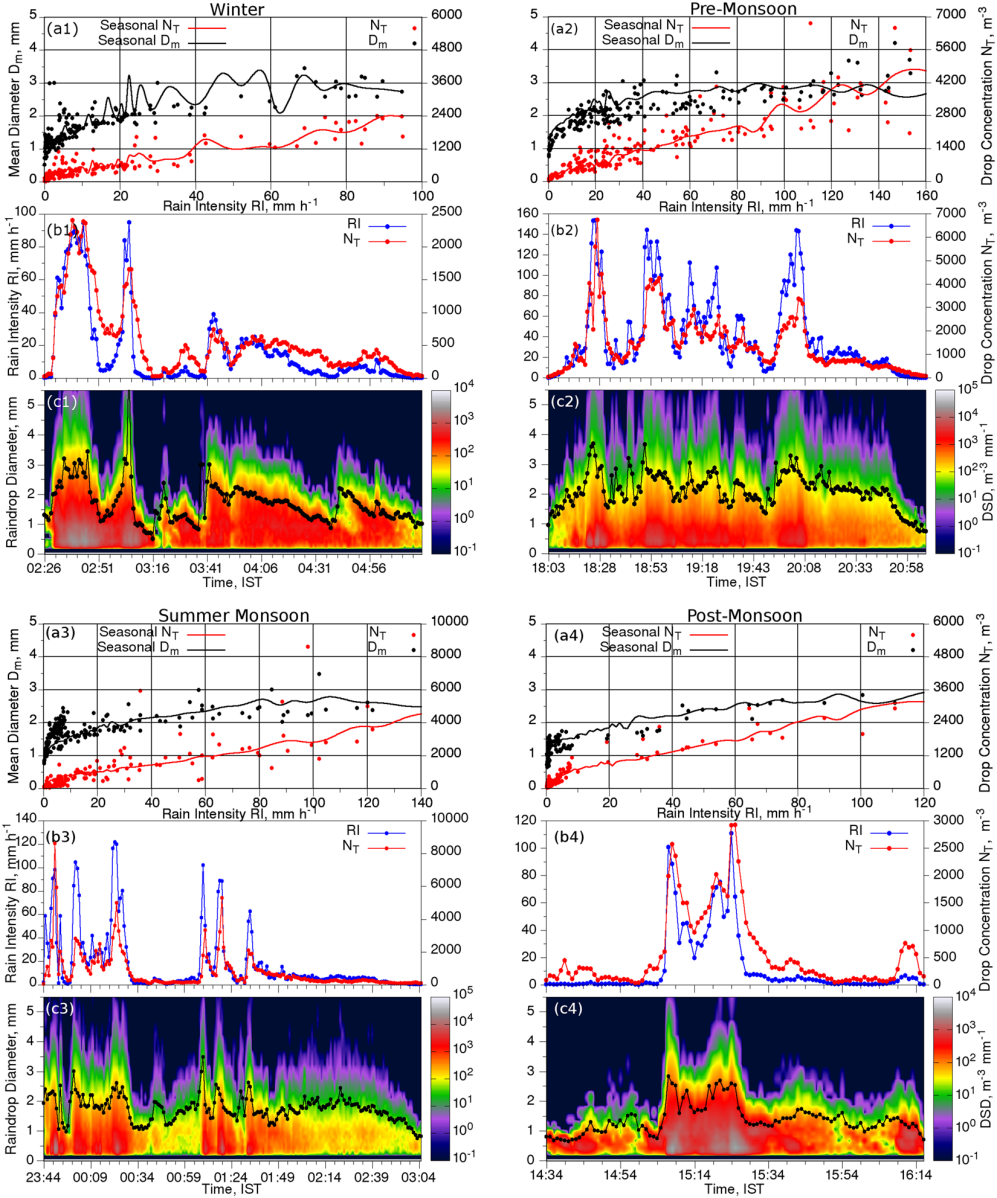
Variation of D_m_ and N_T_ with respect to RI (a1-a4). Temporal variation of RI & N_T_ (b1-b4), and DSD & D_m_ in black line with dots (c1-c4). (Plotted in Gnuplot and combined using Gimp).

The case studies of rain events during different seasons (Fig. 8) are also analyzed and compared with the results discussed based on the average features of RI, N_T_, D_m_ and DSD explained in the earlier sections. These DSD derived microphysical parameters and DSD spectrum are plotted with respect to time of the rain event.

The first case is considered (19–02–2013, 02:20–05:20 hrs) from the winter season with a total duration of 175 minutes and 43.4 mm of rainfall (Fig. 8a1 to c1). The mean (SD) values of RI, N_T_ and D_m_ are 15.9 (26.06) mm h^−1^, 554.67 (548.86) m^−3^ and 1.71 (0.71) mm, respectively. The event consists of two major peaks in the beginning and followed by multiple minute peaks. RI recorded up to 95 mm h^−1^ and N_T_ up to 2400 m^−3^ in the first 30 minutes with D_m_ of about 3 mm and thereafter the event is under the dissipation stage with lower values of RI, N_T_ and D_m_ (Fig. 8b1,c1). This period of the rain event can be considered as convective phase because RI is greater than 10 mm h^−1^ for more than 30 minutes^53^. In the second peak of rain, N_T_ is not reached up to that of the first one with same RI values; however, the D_m_ value is much higher (>5.5 mm) than the first peak indicating the relative contribution of massive drops towards the higher intensity of rainfall. During the dissipation phase, RI and N_T_ show four minor intensifications and corresponding enhancements are also observed in D_m_. During these minor intensifications, RI and N_T_ marked up to 40 mm h^−1^ and 1000 m^−3^, respectively, with an average D_m_ of 2 mm. During the peaks in the first 30 minutes, the rate of growth of RI and N_T_ in the progress stage is simultaneous and gradual, however, during the dissipation of phase of the peaks, the depletion of RI and N_T_ are not concurrent but with a slight delay in depletion of N_T_. This fast (gradual) dissipation in RI (N_T_) is due to lack of raindrops in large drop sizes. This indicates the formation (dissipation) of large (small) raindrops in the convective phase of the rain event. Updraft during convective rain provides more time for the formation of drops, which in turn results in the build-up of large raindrops due to buoyancy^50^. In the dissipating phase of the event, all intensifications are steep, and corresponding weakening are gradual for RI and N_T_. In the five rain types (R1 – R5), DSD matches well with average winter DSD (Fig. 6). Seasonal D_m_ and N_T_ variation with RI from Fig. 7 is also compared with this rain event and found they are in good agreement.

The second rain event (24-04-2012, 18:03–21:07 hrs) is considered from the pre-monsoon season with a duration of 185 minutes and 125.7 mm rainfall (Fig. 8a2–c2). The mean (SD) of RI, N_T_ and D_m_ are 40.77 (38.25) mm h^−1^, 1408.63 (1143) m^−3^ and 2.14 (0.58) mm, respectively. In this event, RI reached up to 155 mm h^−1^ and N_T_ up to 6880 m^−3^. For the first two hours, the phase of the event is convective with abnormal enhancement of the RI. During the peaks in RI, less N_T_ and high D_m_ are observed except for first rain peak. Values of N_T_ and D_m_ are high to provide the highest RI in the case of first peak (Fig. 8c1). However, sharp rises of D_m_ are not observed in this event as we reported during the winter event. The rainfall systems during the season is predominantly convective^37,52^ and therefore, the average D_m_ values are high during this period and it is also confirmed by the high mean values of D_m_ during entire pre-monsoon period. Due to the background high values in D_m_ with less value in SD, a sharp increase in D_m_ may not be registered in this event during the peaks of RI while comparing with the winter rain event. The DSD spectrum of the rain event for the rain types from R1 to R6 follows the same pattern of seasonal mean of DSD spectrum during the winter season. The huge number of small drops and relatively large number of large drops in R5 and R6 is observed in the time series of DSD for this event. The variation of D_m_ and N_T_ during this rain event is also in good agreement with the seasonal variations (Fig. 7). Growth and dissipation of RI and N_T_ are accompanied with accumulation and destruction of raindrop density in large sizes.

The third case is analyzed from summer monsoon (23:44 hrs, 16-09-2017 to 03:05 hrs, 17-09-2017) and is given in Fig. 8a3–c3. The mean (SD) of RI, N_T_ and D_m_ are 17.54 (26.35) mm h^−1^, 787.95 (1100) m^−3^ and 1.75 (0.42) mm, respectively. Maximum recorded RI and N_T_ are 120 mm h^−1^ and 9000 m^−3^, respectively, during this event. Rainfall recorded during this event is 59.05 mm from a total duration of 202 minutes. This rain event has two active phases, the first one is within first 40 minutes and the second one is after 30 minutes of the first one, with three rain peaks. During the entire event, about six rain peaks are observed and these peaks are short lived. During summer monsoon rain event, more N_T_ is involved with less D_m_ for contributing to RI than all other seasons. For different rain types, the observed N(D) in all drop sizes are comparable/similar with the temporal variation of DSD of this event with minor deviations. Seasonal pattern of N_T_ and D_m_ are matching with this rain event (Fig. 8a3). Mean diameter is widely fluctuating for the RI < 10 mm h^−1^ when compared with seasonal relationship. This fluctuation is may be due to the presence of large raindrops in starting stage of the rain event. Seasonal N_T_ is in good agreement with the pattern of N_T_ during the event as seen in Fig. 8c1.

One event from post-monsoon is also analyzed as fourth case (Fig. 8a4–c4). This rain event is on 19-10-2012 from 14:34 hrs to 16:16 hrs with a total duration of 103 minutes and a total rainfall of 25.86 mm. In this event, RI reaches a maximum of 110.84 mm h^−1^ and that for the N_T_ is 2927 m^−3^. The mean and SD of RI is 12.56 and 23.58 mm h^−1^ and that for N_T_ is 580.12 m^−3^ and 696.81 m^−3^. The mean value of D_m_ is 1.4 mm with a standard deviation of 0.65 mm. During the first 30 minutes of the rain event, the intensity is less and thereafter, rain gets intensified with two major rain peaks in the next 30 minutes duration. The RI is less intense with less values of N_T_ during the remaining period and D_m_ is also showing low values during this dissipation period. Small drops are dominated in the initial stage of the rain event and then it transforms to large drops as the event gets intensified along with intensification of N_T_ and D_m_. In the dissipating stage, D_m_ is high due to the presence of large raindrops with less N_T_ while comparing with other seasons and this situation persists till the end of the event. Seasonal DSD is also well matching with the time series of DSD variation of this rain event. The variation of N_T_ during this event matches with seasonal variation of N_T_ for different rainfall intensity ranges, especially for the bins above 20 mm h^−1^. Mean drop diameter of the event also shows similar behavior as that of the seasonal pattern.

Rain events for the case studies are selected to cover low, medium and high intensity ranges of rain with considerable longevity. From these analyzed rain events, pre-monsoon event collected maximum rainfall and correspondingly the microphysical parameters. Summer monsoon rain is characterized by small raindrops along with less D_m_ and SD. During the post-monsoon, D_m_ shows high variability with low mean value. It is also observed from the variation of N_T_ and D_m_ with RI that raindrops are larger after 80 mm h^−1^ due to the convective rain at higher RI. Thus, the observations from case studies are supporting the results from statistical analysis of microphysical parameters, seasonal distribution of DSD in different rain types and variation of N_T_ and D_m_ with RI.

### Distribution of RI, N_T_ and D_m_

To segregate the microphysical features of rain and their seasonal difference at this WG mountain station, the RI, D_m_ and N_T_ are classified into different range bins as shown in Table 2, and their percentage of occurrence and accumulated rainfall in each range bins are also computed. The WG mountain ranges are fragile and significant because of their geographical, environmental, hydrological and meteorological features. Estimation of rainfall from the WG is vital in several fields such as meteorology, hydrology and more particularly in disaster management. Since hazards are mainly related to rain (eg. land slide, flash flood, cloud burst, etc.), it is crucial to understand rain in terms of its microphysics such as RI, N_T_ and D_m_. Seasonal characteristics of rain in terms of its microphysical parameters along with their relative contribution of rainfall to the total rainfall for different ranges are discussed in the next sections.

#### Distribution of Rain types and their contribution to Rainfall

**Figure 9:**
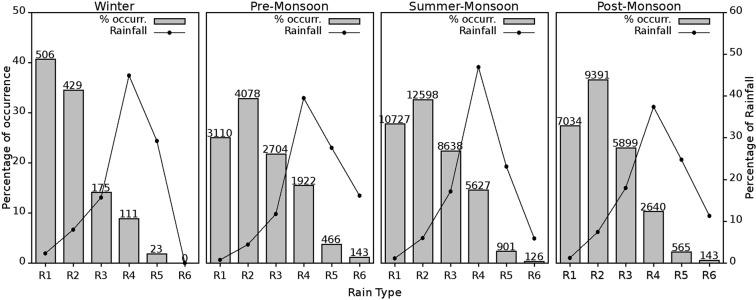
Percentage of occurrence (bar) and relative rainfall contribution (line) to the total rainfall from different rain types in four seasons. The number above each bar indicates the total duration registered. (Plotted in Gnuplot).

Percentage of occurrence in different rain types and their relative rainfall contribution in all the four seasons are given in Fig. 9. The numbers given above each bar in the figure denote the total number of occurrence in each rain category. R2 is the predominant rain type followed by Moderate Drizzle (R1) and then Moderate Rain (R3) in all the seasons except in the winter. During the winter, R1 has the maximum number of occurrences, and it is followed by Light Rain (R2). Even though the occurrence number in each rain type vary, a steady decrease is observed in the percentage of occurrences from R2 to R6 in all the four seasons, while in the winter it is from R1 onwards.

The distribution of rainfall relative contribution is different from the rain type distribution pattern. It is evident that the percentage of occurrence is high in low-intensity (R1 – R3) rain types, but the relative contribution from those types are less to total rainfall. More than 70% of the total rainfall is contributed from the high-intensity (R4 – R6) rain types. R4 registers more than 40% of the total rainfall in all the four seasons followed by more than 25% from R5. More than 45% of total rainfall is from R4 during the winter and summer monsoon, whereas during the pre- and post- monsoons, the contribution from the R4 is less than 40%. R6 contributed more than 10% during the pre- and post- monsoons and this is caused for relative decrement in relative contribution from the R4 type. R5 and R6 are disastrous at a mountain site as they can pour enormous amount of rainfall within a short period. During pre- and post-monsoons, more than 35% of total rainfall is contributed by R5 and R6 and this may lead to floods and flash floods over the study area. The probability of occurrences of these rain types from thunderstorms is high as this site is instrumental in forming thunderstorms^14,34,35^. The severity of the pre-monsoon rainfall is high over this mountain location as manifested with high occurrences of R6 rain category. During the pre-monsoon, the duration (143 minutes out of 12423 minutes) of R6 rain is almost double from that of the post-monsoon season (143 minutes out of 25672 minutes).

#### Distribution of Mean drop diameter and their contribution to Rainfall

**Figure 10:**
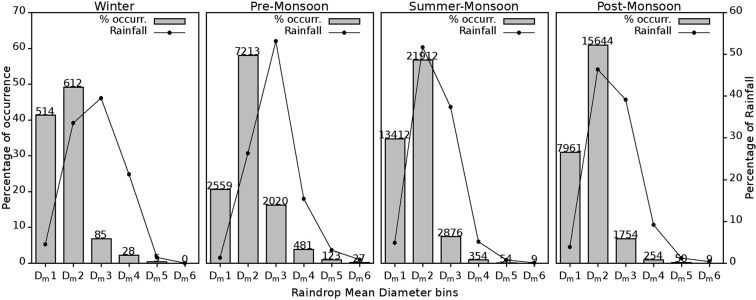
Percentage of occurrence (bar) and relative rainfall contribution (line) to the total rainfall from D_m_ bins in four seasons. The number above each bar indicates the total duration registered. (Plotted in Gnuplot).

Figure 10 demonstrates the percentage of rain occurrence in different D_m_ bins (Table 2) and their percentage of contributions to total rainfall. Seasonal distribution in the percentage of occurrence of D_m_ bins is nearly same in all the four seasons. The frequency of occurrence is high in D_m_2, and the second maximum is in D_m_1 followed by D_m_3 in all the four seasons. However, pre-monsoon marked maximum occurrence percentage in D_m_3 to D_m_6 and least found in D_m_1 compared to the other three seasons. D_m_1 registered its maximum percentage of occurrence during the winter. This increased percentage of occurrence in large drop diameters, particularly in D_m_3 and D_m_4 is well supported by the analysis on variations of N_T_ and D_m_ with RI (Fig. 7).

Relative contribution of rainfall toward the total rainfall from different D_m_ bins is overlaid in the figure as line plot and found that the distribution of contribution is different from that of the occurrence frequency. In the winter and pre-monsoon seasons, maximum rainfall is found in D_m_3 followed by D_m_2 and D_m_4. In the case of summer monsoon and post-monsoon, the contribution of rainfall is maximum from the D_m_2 bin and followed by D_m_3 bin. During the post-monsoon season, relative contribution of rainfall from D_m_4 and D_m_5 is considerably high with more than 10%, however, in these bins contributes only about 6% of the total rainfall in the summer monsoon. Compared to other seasons, summer monsoon received the least percentage of rainfall from D_m_4 to D_m_6 bins, which is well strengthened by the D_m_ and N_T_ variation with respect to RI (Fig. 7) and the respective case study (Fig. 8a3–c3). The argument for the formation of large raindrops in mountain site during the pre- and post-monsoon seasons is evidenced by the high rainfall content from D_m_3 to D_m_6 and D_m_3 to D_m_5, respectively.

From these analysis it is evident that winter and pre-monsoon rains are mostly contributed from the large raindrops (>D_m_3) compared to summer and post-monsoons. During these seasons, this site in the WG is influenced by locally generated, orography driven convective clouds. Because of this orographic convection, effective raindrop formation mechanism will be collision-coalescence, and the probability for the formation of large raindrops is more likely. Moreover, the warm pre-monsoon season is favourable for faster evaporation of smaller drops that present in large numbers has a large surface area to volume index than the large drops^50^. Summing up, because of all these favourable factors large raindrops in the range D_m_3 & D_m_4 even up to D_m_5 & D_m_6 may occur. High relative humidity (RH) and low atmospheric temperature in the summer monsoon^54^ restrict evaporation of small drops and the accumulated rainfall peaks in D_m_2 category. The post-monsoon season is also reported high RH in the atmosphere, thus the formation mechanism of rain is principally convective, and it leads to the development of large raindrops. In hydrological view, considering the kinetic energy of large-sized raindrops, they are effective in detaching soil particles by splash^31,55^. So, these large sized drops initiate landslides, flash flood or such disasters in this unstable sloppy terrain of the WG.

#### Distribution of Raindrop number concentration and their contribution to Rainfall

**Figure 11:**
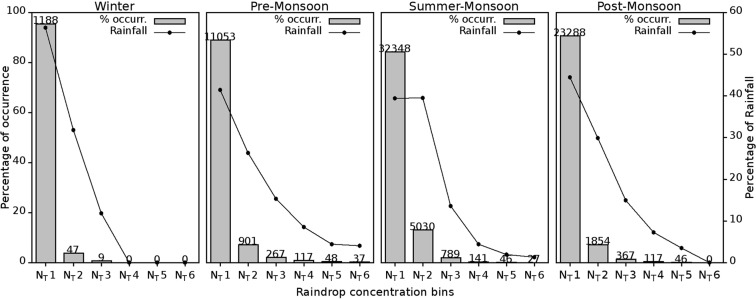
Percentage of occurrence (bar) and relative rainfall contribution (line) to the total rainfall from N_T_ bins in four seasons. The number above each bar indicates the total duration registered. (Plotted in Gnuplot).

Figure 11 describes the percentage of occurrence in different N_T_ bins and associated percentage of rainfall as tabulated in Table 2. Frequency of occurrence of N_T_ and their relative rainfall contribution are dominated in N_T_1 for all the seasons, except for the summer monsoon, there both N_T_1 & N_T_2 are dominated, which followed by a steep decrease in subsequent bins. In the summer monsoon, N_T_1 and N_T_2 bins received nearly same rainfall percentage and contributed about 80% of total rainfall from 10–2000 m^−3^ drops range. Among the seasons, N_T_1 registers less percentage of occurrences (84%) during the summer monsoon and high in the other three seasons with an average of 91.72%. In N_T_2, high percentage of occurrence (13.1%) is in the summer monsoon, and in all other seasons, the mean is 6.1%. This difference is also found in the relative contribution of rainfall. The 13% hike in the percentage of occurrence in N_T_2 seems to be adjusted by a reduction in N_T_4 and N_T_5 during the summer monsoon. During the pre-monsoon, there is a hike in the contribution of rainfall percentage from higher N_T_ bins. Compared to other seasons, pre-monsoon rainfall records maximum from N_T_3 to N_T_6 bins.

### Rain Intensity–Duration analysis

**Figure 12:**
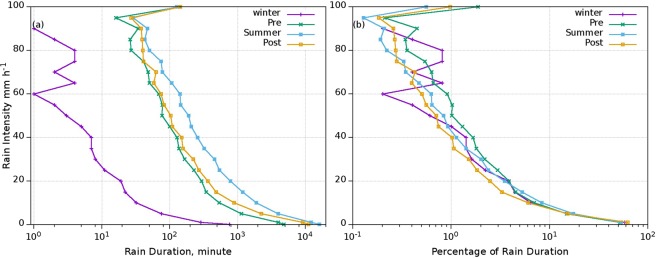
(**a**) Intensity–duration and (**b**) Intensity–percentage of rain duration in four seasons. (Plotted in Gnuplot and combined using Gimp).

Rain Intensity–Duration is analyzed for all the seasons for the seven-year period from the study location and is given in Fig. 12. Almost similar pattern is observed in four seasons except for the winter. During the winter, rain availability is less compared to other seasons and it is also reflected in the rain duration (Fig. 12a). More rain duration exists in the summer monsoon followed by the post- & pre-monsoons and winter seasons. In the duration range from 100–1000 minutes, the RI varies from 18 to 65 mm h^−1^ during summer monsoon, but it for post-, pre- and winter monsoons are 10–50 mm h^−1^, 5–45 mm h^−1^ and 0–5 mm h^−1^, respectively. For rainfall intensity greater than 95 mm h^−1^, the duration is decreasing with increase of intensity.

To normalise the rainfall duration with respect to seasons, duration is converted to its percentage for the respective season in order to remove the quantitative bias in duration of rainfall and it is plotted with respect to RI in Fig. 12(b). In all the seasons, RI up to 20 mm h^−1^, duration percentage is similar, which occurred more than 90% of the total duration. During the pre-monsoon, RI in the range of 20–100 mm h^−1^ registered a higher percentage in duration followed by the summer monsoon. From 70 mm h^−1^ of RI, post-monsoon preceded summer monsoon after pre-monsoon in the percentage of total rain duration. Even though only slight variations are observed in log scale duration, it must note that the number of days is different for these seasons and the small percentages matters in log intervals, especially in the summer monsoon.

## Conclusions

Seasonal variations of different features and microphysical properties of rain are investigated at a tropical mid-land mountain slope site. The measurement site, Braemore, located at southern Peninsular India receives a good amount of rainfall throughout the year except in the winter. In the seven year study period, the station registered a total rainfall of 9264.77 mm from 3551 rain events, which consists of total duration of 1299.3 hours. Rainfall received and time taken per event and their seasonal variations are described in the annual distribution of mean rainfall and duration per event. Variations in microphysical properties of rain are investigated in terms of DSD and its integral parameters during the winter, pre-monsoon, summer monsoon and post-monsoon seasons.

Comparison of PARSIVEL disdrometer data with conventional manual rain gauge data shows that they are incredibly consistent with a correlation of more than 0.99, which is significant at 99.99% confidence level. The annual cycle of rainfall showed a bimodal variation with a primary peak during the summer monsoon months and secondary peak in pre-monsoon period at this WG site. Since the site is reported to be instrumental in thunderstorm formation, copious rainfall during thunderstorm periods (pre- and post- monsoons) indicates the influence of orographic convection.

Main features in the monthly distribution of rain duration and rainfall per event, rain duration and rain events, trace rain duration and DSD derived microphysical parameters at this mountain slope are,

- The winter is the least rainfall season with more number of large drops and less number of small drops, particularly in February.
- During pre-monsoon, less number of long intense rain events with large raindrops along with less number concentration (N_T_) is observed. The variability in RI, D_m_ and N_T_ are found to be high during the season.
- During the summer monsoon season, the number of short-duration mild rain events is high with large number of small drops with relatively low variability in all rain parameters.
- Post-monsoon rain characterized long duration, but less intense events with a few large raindrops compared to summer monsoon.
- The duration of trace rains are high in March and July and then it decreases steadly from July to December.

From the variation of N_T_ and D_m_ with RI, it is observed that large drops are formed during the winter and pre-monsoon within 80 mm h^−1^ of RI, whereas it is above 80 mm h^−1^ during the post-monsoon season. Massive raindrops are found in the RI of 80–100 mm h^−1^ and it decreases thereafter during the summer monsoon, which also suggests the number of small-sized drops is more at high RI. Seasonal DSD spectrum showed more number of large drops and less number of small drops of different RI bins during pre- and post- monsoons than that during the summer monsoon season. In the case of winter, the similar spectrum is also observed in Violent rain type, which indicates the initiation of orographic convection in this mountain slope with less magnitude and it is also important to note that the Very Violent rain type is completely absent during this season. These inferences specify that this mountain slope experiences orographic convection and associated rainfall in February, pre- and post- monsoons, whereas it is less in the summer monsoon.

Observations from the case studies are supporting the results from statistical analysis of microphysical parameters, seasonal distribution of DSD in different rain types and variation of N_T_ and D_m_ with RI. From the analyzed sample rain events, maximum values of rainfall and other microphysical parameters are found in the pre-monsoon season. The basic characteristics of small raindrops are indicated by less D_m_ and SD during summer monsoon rain event. In post-monsoon rain event, less mean and high SD in D_m_ indicate small raindrops with high variability. It is also observed from the variation of N_T_ and D_m_ with RI that raindrops are large above 80 mm h^−1^ due to the convective rain at high RI during the post-monsoon season. From the distribution of microphysical parameters and their contribution to the total rainfall, the maximum frequency of occurrence is registered in the R2 bin among the six rain types in all the four seasons except in the winter. The high occurrence (double that of post-monsoon) of R6 rain type manifest the intensity of pre-monsoon rainfall at this mountain slope. R5 and R6 rain types contributed more than 35% to the total rainfall during pre- and post- monsoons and this indicates the presence of thunderstorms formation^14,34,35^. During winter and pre-monsoon rains, the rainfall is contributed primarily from the large raindrops (>D_m_3), however, during the summer and post-monsoons, it is from D_m_2 bin onwards. Among N_T_ bins, for all the seasons N_T_1 registers maximum occurrence and rainfall percentages. N_T_2 recorded rainfall percentage nearly the same as N_T_1 in the summer monsoon and this also supports large number of raindrops in the season. From Intensity-Duration analysis, it is noticed that more rain duration exists in the summer monsoon followed by post- & pre- monsoons and then winter. It is also inferred that more than 90% of rain duration is from RI less than 20 mm h^−1^ in all the seasons.
